# FK-means: automatic atrial fibrosis segmentation using fractal-guided K-means clustering with Voronoi-clipping feature extraction of anatomical structures

**DOI:** 10.1098/rsfs.2023.0033

**Published:** 2023-12-15

**Authors:** Marjan Firouznia, Markus Henningsson, Carl-Johan Carlhäll

**Affiliations:** ^1^ Unit of Cardiovascular Sciences, Department of Health, Medicine and Caring Sciences, Linköping University, Linköping, Sweden; ^2^ Center for Medical Image Science and Visualization (CMIV), Linköping University, Linköping, Sweden; ^3^ Department of Clinical Psychology in Linköping, Department of Health, Medicine and Caring Sciences, Linköping University, Linköping, Sweden

**Keywords:** fibrosis segmentation, left atrium, pulmonary veins, clipping, K-means, deep learning

## Abstract

Assessment of left atrial (LA) fibrosis from late gadolinium enhancement (LGE) magnetic resonance imaging (MRI) adds to the management of patients with atrial fibrillation. However, accurate assessment of fibrosis in the LA wall remains challenging. Excluding anatomical structures in the LA proximity using clipping techniques can reduce misclassification of LA fibrosis. A novel FK-means approach for combined automatic clipping and automatic fibrosis segmentation was developed. This approach combines a feature-based Voronoi diagram with a hierarchical 3D K-means fractal-based method. The proposed automatic Voronoi clipping method was applied on LGE-MRI data and achieved a Dice score of 0.75, similar to the score obtained by a deep learning method (3D UNet) for clipping (0.74). The automatic fibrosis segmentation method, which uses the Voronoi clipping method, achieved a Dice score of 0.76. This outperformed a 3D UNet method for clipping and fibrosis classification, which had a Dice score of 0.69. Moreover, the proposed automatic fibrosis segmentation method achieved a Dice score of 0.90, using manual clipping of anatomical structures. The findings suggest that the automatic FK-means analysis approach enables reliable LA fibrosis segmentation and that clipping of anatomical structures in the atrial proximity can add to the assessment of atrial fibrosis.

## 1. Introduction

Cardiac magnetic resonance imaging (MRI) is a non-invasive and versatile tool that adds to the assessment of cardiac structure, function and tissue characteristics in atrial fibrillation (AF) [1]. AF leads to morphological changes in the left atrium (LA) such as increased atrial volume and shape differences [2]. This remodelling process is associated with an increase in fibrosis of the LA wall, which, in turn, leads to further structural and functional changes in the atrium. Late gadolinium enhancement (LGE) MRI is widely used for visualizing and quantifying atrial fibrosis [3–5]. The assessment of fibrosis in the LA using LGE imaging offers valuable insights into the management of AF patients [6], for instance predicting AF recurrence after ablation procedures [3]. However, accurately assessing fibrosis within the LA wall remains challenging in clinical practice. Recent studies have focused on accurately segmenting and quantifying fibrosis in the LA wall by excluding the mitral valve (MV), pulmonary veins (PVs) and left atrial appendage (LAA) to avoid misclassification of non-fibrotic regions as fibrotic. Sim *et al*. [7] proposed a pipeline for processing LGE-MRI scans, which requires manual segmentation of the LA and labelling of anatomic structures. Tobon-Gomez *et al*. [8] proposed a pipeline for LA segmentation, involving generating a surface mesh, defining a local coordinate system and obtaining a consistent definition of the PV ostia. Razeghi *et al*. [9] proposed an automatic method for assessing atrial fibrosis using a multi-label convolutional neural network (CNN), involving a clipping and truncation of the PVs and MV regions on CE-MRA (contrast-enhanced magnetic resonance angiography) and LGE-MRI. The method combines automatic ostia localization and truncation techniques to assist in the manual segmentation process of LA, PVs and MV. The CNN model was trained using manual segmentations of the anatomical structures as part of a multi-label learning approach.

The aim of the study was to develop and validate an automatic approach for segmenting LA fibrosis from LGE-MRI images, while excluding MV, PVs and LAA. High FD and tortuosity values are used to represent the morphology of the LA wall. The output of the method is a region referred to as the Clipped_LA. To accurately classify fibrotic regions in the LA, FK-means used a hierarchical adaptive K-means clustering method that exploits differences in FDs. The proposed modification of the 3D K-means algorithm incorporates the K-means++ initialization method to improve clustering accuracy.

## 2. Related works

Fibrosis segmentation and quantification methods can be classified into threshold-based and classification-based techniques [10]. Threshold-based methods, such as Otsu thresholding, hysteresis thresholding and constrained watershed segmentation [11,12] are limited in handling variations in morphology, brightness, resolution, contrast, signal-to-noise ratio, inversion time and surface coil intensity [13]. Classification-based methods such as K-means clustering and random forest classification have also been employed for fibrosis segmentation [11,14].

Several deep learning-based methods have been developed for LA scar segmentation and quantification. Yang *et al*. [15] used superpixel over-segmentation for feature extraction and a supervised classification step via stacked sparse auto-encoders for LA scar segmentation. Li *et al*. [16] proposed a hybrid approach that combined a graph-cuts framework with CNNs for automatic scar segmentation, and extended their work with a multi-scale CNN to learn local and global features simultaneously. Chen *et al*. [17] used multi-task learning for simultaneous LA and scar segmentation, but did not explicitly learn the spatial relationship between the two regions. Li *et al.* [18] proposed the use of multi-scale patches for scar quantification, but this approach is computationally expensive and limits end-to-end training with optimization on the whole graph. To overcome these limitations, AtrialJSQnet [20] was developed using deep neural networks to jointly segment and quantify blood cavity and scars of LA from LGE-MRI, incorporating a novel shape attention technique and a spatially encoded loss to explicitly use spatial relationships and incorporate spatial information without network modifications [19].

In a recent study, 3D late gadolinium enhanced Dixon MRI was found to be a feasible method for simultaneous assessment of LA fibrosis and epicardial adipose tissue (EAT) [21]. In the current study, the data and labels obtained from this former study are considered for LA fibrosis segmentation based on the valve reference method [22] to generate a ground truth for the current work.

## 3. Methodology

**Figure 1.**
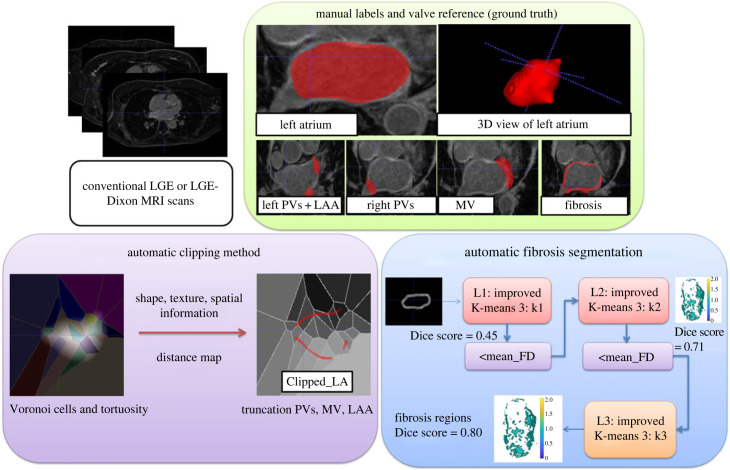
An automatic method for estimating left atrial (LA) fibrosis from LGE and LGE-Dixon MRI scans, which involves both clipping and clustering steps. Manual labels and ground truth are used, shown in the green panel. In the clipping step, image processing techniques, shown in the purple panel, are applied to clip the mitral valve (MV), pulmonary veins (PVs) and left atrial appendage (LAA) from the LA. To achieve this, shape and texture features are extracted from the Voronoi cell related to the LA wall, resulting in Clipped_LA (LA wall without PVs, LAA and MV). The fibrosis segmentation clustering step, depicted in the blue panel, incorporates an enhanced 3D K-means++ algorithm as a novel machine learning method with three levels of clustering (L1–L3). The stopping metric employed is the mean FD, where clustering can be performed continuously to improve the Dice score. We use the mean FD of the resulting fibrosis region as a decision criterion for determining whether to proceed to the next level of clustering or increase the number of clusters from 3 to k1, k2 and k3 in the respective levels.

The FK-means method is a machine learning method based on K-means for the automatic segmentation of fibrosis in the LA, achieved by clipping the MV, PVs and LAA. The proposed clipping step used an automated truncation approach that incorporates texture and shape features to characterize the morphological and textural variations of anatomical structures. This approach employed a Voronoi diagram around the LA wall to clip and truncate anatomical structures from the image in an automated manner. The Clipped_LA wall was then subjected to a classification step for fibrosis segmentation. The proposed method is entirely automatic, and an overview of the proposed methods is shown in figure 1.

### 3.1. Clinical magnetic resonance imaging data

A total of 35 patients with a clinical indication for cardiac MRI were included in the study. Seven patients underwent conventional LGE imaging (subgroup 1) and 28 patients underwent LGE-Dixon imaging (subgroup 2). All patients had either AF, other cardiac diseases, or no cardiac disease, and none had undergone ablation. The MRI scans were performed using a 1.5 T MRI scanner (Philips Healthcare, Best, The Netherlands) equipped with 28-channel receive coils. The LGE scans had a field-of-view (FOV) of 320 × 320 × 120–140 mm^3^, and a spatial resolution of 1.25 × 1.25 × 2.5 mm^3^.

### 3.2. Manual labelling and reference generation

To assess the accuracy of the current automated segmentation methods for identifying LA fibrosis, the valve reference method used by Skoda *et al*. [21] was employed as ground truth for validation. This method relies on a valve intensity to identify fibrosis as a valve reference model. To ensure accurate segmentation of the anatomical structures, two readers (M.H. with 15 years of experience in cardiac MRI and I.S. with 5 years of experience in cardiovascular imaging) independently confirmed the ground truth and manually labelled the anatomical parts using the same methodology as described by Skoda *et al*. [21]. This approach allowed us to compare the performance of the current automated segmentation methods against an established reference method. The LA myocardium was segmented using the Medical Imaging Interaction Toolkit (MITK), while MATLAB R2021b was used for automatic fibrosis quantification. Figure 1 shows the four manual labels of MV, left PVs, right PVs and LA.

### 3.3. Voronoi-based clipping of anatomical structures

**Figure 2.**
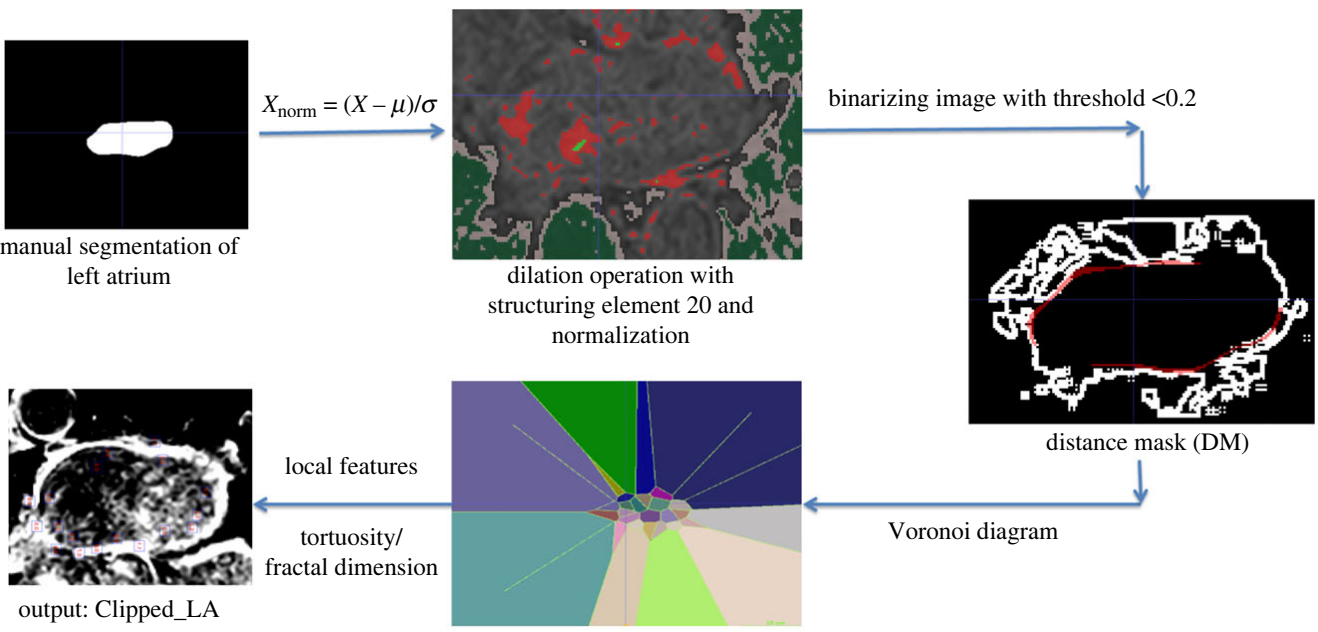
The Voronoi diagram-based clipping approach. The method involves binarizing the input image using a delineation operation, followed by extraction of the distance map and identification of candidate pixels and Voronoi cells. Features including FD and tortuosity are then extracted from the relevant Voronoi cells. Finally, the differences in FD and tortuosity between connected neighbouring Voronoi cells are calculated to identify the regions related to the anatomical structure. Left atrium (LA) refers to the atrial cavity plus the atrial wall.

The clipping method aims to improve segmentation by considering texture information from surrounding organs. The proposed Voronoi diagram-based approach truncates regions associated with the MV, PVs and LAA. As shown in figure 2, the valve's texture resembles a smooth-edged blood pool, making it challenging to distinguish from the LA blood pool. Therefore, the main goal is to find the clipped-LA using this approach. Texture information provides valuable insights for MV, PVs and LAA detection. The clipping method consists of four steps: distance map extraction, Voronoi diagram generation, feature extraction and truncation.

The process starts with the application of a dilation operation based on an LA segmentation using a disc-shaped structuring element with a radius of 20 pixels. Let *X* represent the input image and *R* the segmented blood pool region. Normalization is performed by calculating the mean (*μ*) and variance *σ*^2^ of pixel intensities within the region. The LGE image's pixel intensities can be normalized using $X_{\mathrm{norm}}\;=\;(X\;-\;\mu )\;/\sigma$. Binarization of the normalized image *X*_norm_ is done using a threshold value of 0.2, where intensities greater than or equal to 0.2 are set to 1, and those less than 0.2 are set to 0.

Distance map generation is used to identify candidate pixels related to MV, PVs and LAA based on the LA wall. The distance of each pixel of binarized image from LA wall is calculated to remove distant pixels that are not part of the LA wall. This is important because distant pixels can indicate the appearance of the structures. The distance between the binarized image and the LA wall edge is determined using a filtering method. A diagonal kernel is used to each slice in 2D using the conv2 function to find a distance matrix. The aim of the FK-means method is to generate Voronoi diagrams based on the principles of fractal geometry [23].

To achieve this, Voronoi cells are used to localize and extract features from related regions. By analysing the distance map, the exact location of the MV, PVs and LAA could be identified. However, to avoid selecting unrelated regions, Voronoi cells were considered to localize areas that were distant from the distance map. This approach enabled the identification and extraction of features from regions specifically related to MV, PVs and LAA.

In the last step, FD and tortuosity features were extracted to represent the regions related to the anatomical structure. Voronoi cells were used to consider each region locally, and the features were extracted from the related vortex that was far from the distance map. High FD values indicated a fibrotic region, which is usually related to the MV.

The tortuosity of a snake-like shape can be determined by calculating the ratio of the actual longest spine distance (L) to the straight line distance between the endpoints (D). To compute L, the snake-like shape needs to be segmented, and the spine, representing the central line of the shape, should be identified. This can be achieved by applying morphological operations and skeletonization. Once the spine is obtained, its length represents L. The straight line distance between the endpoints can be calculated using the Euclidean distance. Additionally, the average width of the shape can be determined by measuring the width at multiple points along the spine and taking their average. To summarize, the tortuosity and average width of a snake-like shape are computed by segmenting the shape, finding the spine, calculating L and D for tortuosity, and measuring the width at various points for the average width. The tortuosity feature provided information on whether there was another organ or LA, and if there was, a branch was detected. High tortuosity values indicated the related regions of PVs. Based on empirical observation, if the difference in FD between a current Voronoi cell and its connected neighbouring Voronoi cell is 0.7 and the difference in tortuosity between them is 1, it indicates a relationship between these cells. Based on the analysis, Voronoi cells with a difference in FD of 0.7 and a difference in tortuosity of 1 are closely related to the MV and PVs. The final output is the Clipped_LA without the MV, PVs and LAA.

### 3.4. Fibrosis segmentation using K-means-based derived fractal analysis method

FK-means method uses a hierarchical adaptive K-means clustering approach as a machine learning algorithm to classify fibrosis pixels on Clipped_LA, incorporating automatic tuning parameters. The K-means algorithm, widely used in clustering and segmentation tasks, forms the basis of the approach. A modification to the 3D K-means algorithm is introduced by integrating the K-means++ initialization method. The main objective is to incorporate the FD into the clustering algorithm for accurate fibrosis segmentation. To calculate the FD map of each pixel in the LGE image, the differential box-counting method suggested by Al-Kadi & Watson [23] is employed. The FD estimation is based on the measurement points of log(1/*r*) and log(*Nr*(*x*, *y*)), where *Nr*(*x*, *y*) denotes the number of boxes required to cover the neighbourhood of the pixel. The calculation of *Nr*(*x*, *y*) follows equation (3.1):

3.1 $$Nr(x,y)=\frac{R^{2}}{r^{2}}\times \;\left(\frac{Mr(x,y)-mr(x,y)}{r}\right)+\;1,$$

where $Mr(x,y)=\underset{\left(u,v)\in wr(x,y\right)}{\max}I(u,v)$ and $mr(x,y)=\underset{\left(u,v)\in wr(x,y\right)}{\min}I(u,v)$ with 2 < *r* < *R*. Then, FD(*x*, *y*) is estimated as the slope of a least-squares regression line on the measurement points of log(1/*r*) and log(*Nr*(*x*, *y*)). In the experiments, it was found that selecting a small value for *R* resulted in kernels that did not encompass enough surrounding pixels to accurately characterize the texture through FD. On the other hand, selecting *R* too large resulted in kernels that covered very large regions and did not provide significant texture information.

The modified 3D K-means algorithm incorporates the FD distance metric with penalty terms to encourage the centroids to be close to both their assigned data points and the mean FD of their assigned data points in the previous iteration. The centroid for each cluster is then recalculated based on the mean position of the data points in the cluster. The mean distance between each data point and its assigned centroid is calculated and used as the penalty term for the distance metric in the next iteration. Specifically, the distance between a data point *x* and a centroid *c* is defined as

3.2 $${\begin{array}{rl} & d(x,c)=\;\mathrm{sqrt}({\left\|x-c\right\|}^{2}+\beta *\|\mathrm{FD}(x)-\mathrm{FD}(c)\| \end{array}}^{2}+\gamma *\|\mathrm{FD}(x)-\mathrm{mean}(\mathrm{FD}(c_{i})){\|}^{2}),$$

where \Vert\cdot\Vert is L2 norm to calculate Euclidean distance between data point *x* and centroid *c* in 3D space, FD(*x*) is the FD of data point *x*, FD(*c*) is the FD of centroid *c*, mean(FD(*c_i_*)) is the mean FD of the data points in cluster *c_i_*, *β* = 0.1 and *γ* = 0.5 are penalty parameters. The algorithm repeats these steps until the centroids converge. Alternatively, the process stops if the maximum number of iterations is reached. The motivation behind this method is to improve the clustering performance of the traditional K-means algorithm by incorporating FD as a feature in the distance metric. FD is a measure of the complexity or irregularity of an object, and in the context of image analysis, it can capture texture information that is not captured by traditional pixel-based features. By penalizing the assignment of dissimilar data points to the same cluster based on their FDs, this method aims to cluster pixels with similar textures together while also considering the spatial relationships among the pixels.

Clustering is performed at three different levels with varying numbers of clusters: 2–30 for the first level, 2–5 for the second level and 2–3 for the third level. After each level or changing the clustering parameters, the fibrosis region is identified as the cluster with the maximum fractal dimension, and the mean and variance of the fractal dimensions of the fibrosis region are calculated. To incorporate the stopping metric in the proposed clustering approach, two threshold parameters are introduced, *τ*_1_ and *τ*_2_, for mean and variance, respectively, to detect when further clustering is no longer necessary. Specifically, the mean and variance of the fractal dimensions of the cluster are checked, along with the maximum FD for each level and cluster. If these values significantly differ from the mean and variance of the previous level or cluster, the process of increasing the number of clusters is stopped, and the process then moves on to the next level. The stopping metric for the mean fractal dimension can be formulated as

3.3 $$|\mathrm{mean}({\mathrm{FD}}_{i})-\mathrm{mean}({\mathrm{FD}}_{i-1})|>=\;\tau_{1},$$

where mean(FD*_i_*) is the mean fractal dimension of cluster with high FD of *i*th cluster/level, and mean(FD*_i_*_−1_) is the mean fractal dimension of the previous cluster. The stopping metric for the variance of the fractal dimension can be formulated as

3.4 $$|\mathrm{var}({\mathrm{FD}}_{i})-\mathrm{var}({\mathrm{FD}}_{i-1})|>=\;\tau_{2},$$

where var(FD*_i_*) is the variance of the fractal dimension of cluster *i*, and var(FD*_i_*_−1_) is the variance of the fractal dimension of the previous cluster. If either of these stopping metrics is satisfied, the clustering process for the current level is terminated, and the fibrosis region is identified as the cluster with the maximum fractal dimension. A high-level description of the algorithm is explained in algorithm 1.

**Table:** 

**Algorithm 1.** The hierarchical clustering method.
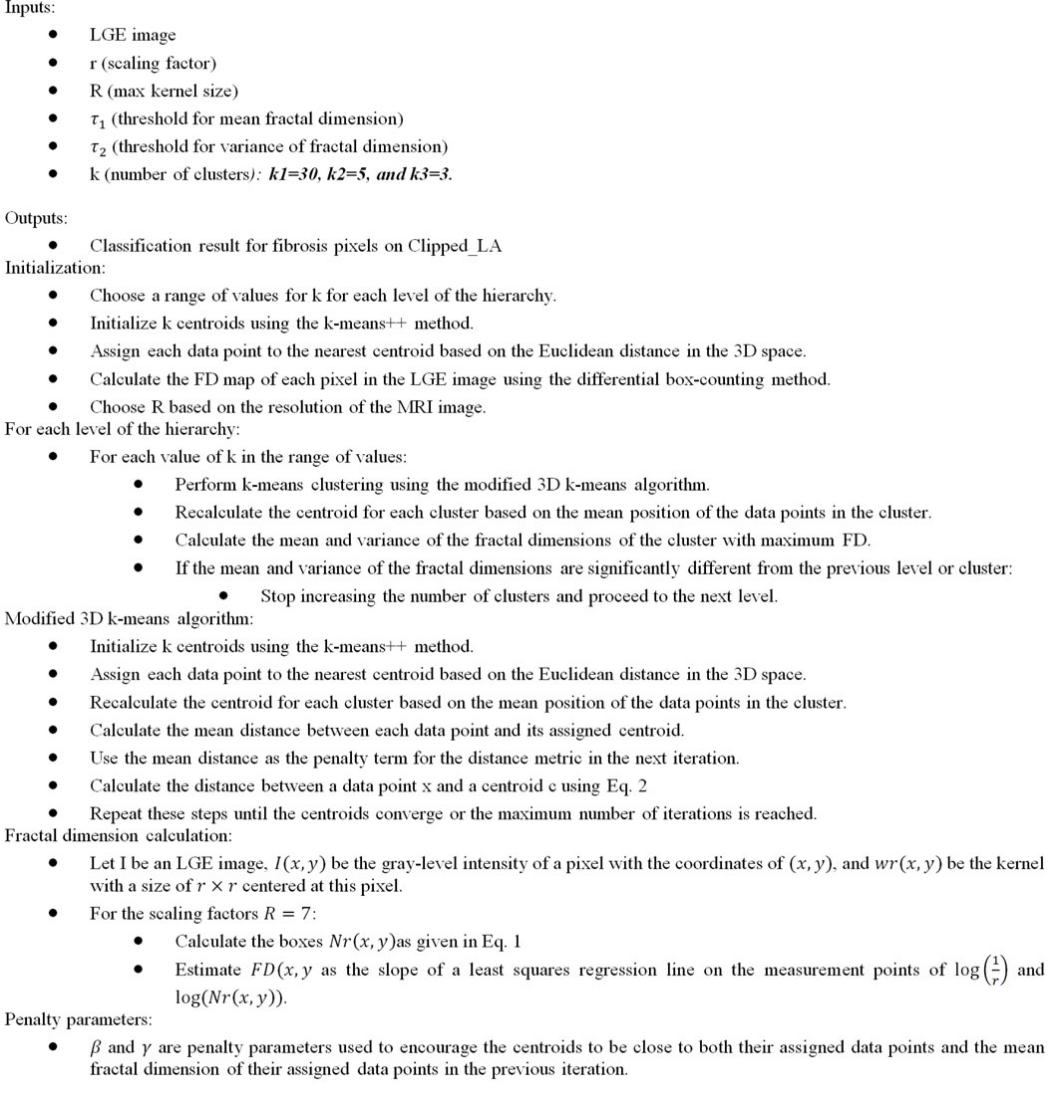

### 3.5. Comparative analysis: deep learning and 3D region growing methods

In this study, the proposed automatic machine learning-based method for fibrosis segmentation was compared with a deep learning-based approach as well as a traditional 3D region growing method. The deep learning method involved implementing two 3D UNet models for fibrosis segmentation and also automatic clipping of anatomical structures. The MONAI library is used, which is a PyTorch-based open-source framework for deep learning in medical imaging. Specifically, a 3D UNet architecture is implemented, with modifications to the number of filters and layers to better suit the fibrosis segmentation task. Additionally, various data augmentation techniques were applied, such as random rotation, scaling and flipping, to increase the robustness of the model and to prevent overfitting.

In addition to the deep learning-based approach, also a more traditional 3D region growing method was employed for fibrosis segmentation. This technique used a seed point within the LA region and iteratively expanded the segmentation based on predefined criteria, incorporating neighbouring voxels with similar characteristics. Also, for the clustering step, a 2D K-means MATLAB function was employed to partition the data into distinct groups based on similarity.

### 3.6. Validation and repeatability analyses

**Figure 3.**
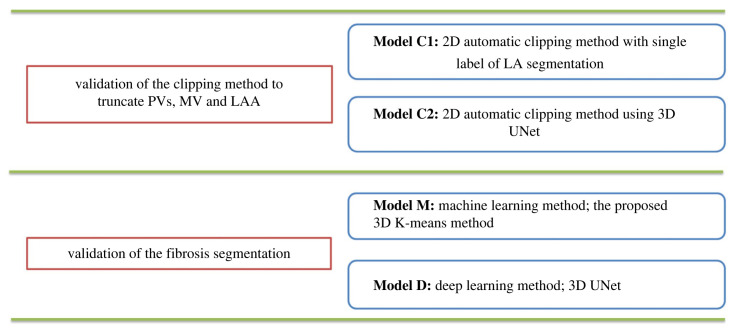
Two steps for validation using three models C, M and D. Models C1 and C2 were used to validate the performance of the clipping part using the Voronoi-based method and the deep learning method, respectively. To validate the quantification of fibrosis segmentation, two models M and D were used, the proposed machine learning and deep learning-based methods, respectively.

In the current study, different models were applied on 35 datasets, as illustrated in figure 3, to validate the performance of the proposed analysis approach. Four models were used to compare the results with the ground truth labels. Model M was used to evaluate the performance of the proposed K-means clustering method. By employing Model D, a 3D UNet model is trained to assess the performance of fibrosis segmentation. Models C1 and C2 were used to assess the clipping method. To evaluate the performance of the clipping and clustering steps, we considered a combination of models: C1, representing the Voronoi clipping model, C2, representing the 3D UNet model as clipping method and D and M, representing the 3D UNet and K-means models for fibrosis segmentation, respectively. Additionally, to evaluate the agreement and repeatability between two manual segmentations, two readers (M.H. and I.S.) provided two sets of manual segmentations for both MV and PVs [21], which were then clipped manually to obtain two Clipped_LA from each set of segmentations. The repeatability analysis was performed using SPSS.

### 3.7. Parameters

The parameter settings used for tortuosity analysis included the disc size used in the dilation operation (20), the minimum branch length used in skeletonization (4), and the fact that the largest blob in the binary image is used for analysis. For fractal analysis, a scaling factor of 7 is used. For clustering step, the stopping thresholds *τ*_1_ = 0.1 and *τ*_2_ = 0.05 are considered. The hyper-parameters of deep learning models included the use of a learning rate scheduler with an initial learning rate of 0.001, weight decay of 0.0001 and stochastic gradient descent optimizer. Also various data augmentation techniques are applied during the training process, such as spatial cropping, orientation adjustment and spacing normalization, to enhance the robustness of the model against different image variations. The use of divisible padding was also employed to ensure the compatibility of the input image and the model's architecture. The evaluation metrics used in this study provide a comprehensive assessment of the accuracy of estimated fibrosis compared to the ground truth. The Dice index measures the overlap between the segmented region and the ground truth, while precision and recall evaluate the model's ability to avoid false positives and false negatives. The evaluation of segmentation accuracy involved two key metrics: the average symmetric surface distance (ASSD) and the maximum symmetric surface distance (MSSD). ASSD quantifies the average distance between the estimated fibrosis and the ground truth, while MSSD represents the maximum distance. Additionally, in order to mitigate potential issues arising from small noisy segmentations, the traditional Hausdorff distance (HD) was adapted by using its 95th percentile, denoted as H95, instead of the maximum distance. These metrics collectively served as critical tools to gauge the accuracy of our segmentation results.

## 4. Results

### 4.1. Validation of the proposed clipping method to truncate mitral valve, pulmonary veins and left atrial appendage

**Figure 4.**
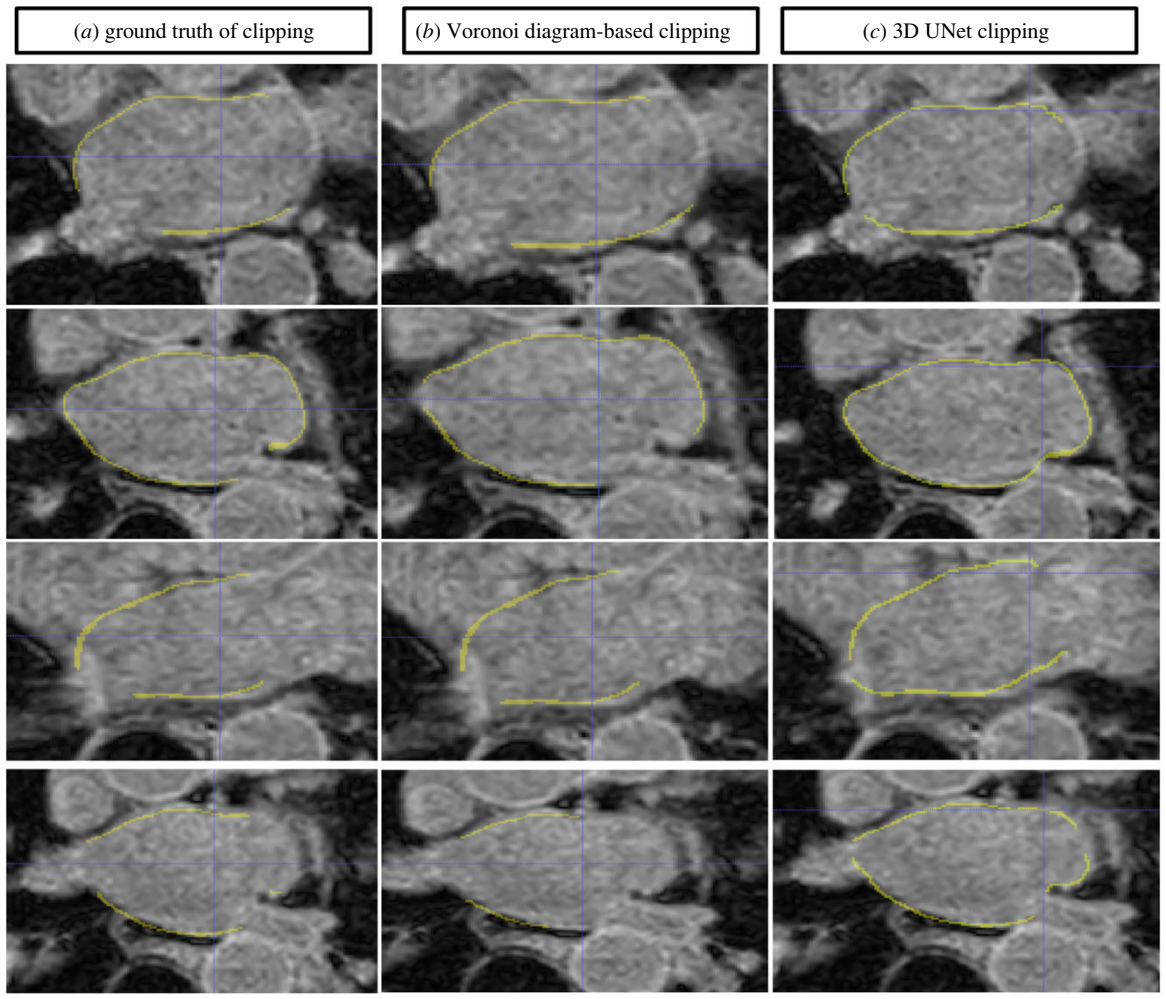
Comparison of clipping methods. Clipped_LA in yellow. (*a*) The ground truth. (*b*) The Voronoi diagram-based clipping approach. (*c*) The deep learning clipping method.

The performance of the Voronoi diagram-based clipping approach was evaluated by comparing the results obtained using the 2D automatic clipping method with the manual clipping performed by readers. The proposed automatic Clipped_LA segmentation method, model C1, showed promising results in comparison to manual clipping with a Dice score of 0.75 on 35 MRI datasets. Figure 4 demonstrates the results and performance attained by the proposed method for Clipped_LA, thereby emphasizing the effectiveness of clipping.

### 4.2. Validation of the fibrosis segmentation method

In this subsection, the performances of the proposed automatic machine learning-based method (K-means-based method) and traditional method (region growing) are compared for fibrosis segmentation.

The results obtained from the experiments are summarized in table 1. In terms of fibrosis segmentation using the proposed 3D K-means algorithm, the Dice scores were 0.90 and 0.76 for manual and the proposed clipping methods, respectively, when applied to 28 LGE_Dixon images. For 7 LGE images, the scores were 0.83 and 0.72 for manual and the proposed clipping methods, respectively. Comparatively, the 2D K-means algorithm yielded lower scores, with 0.54 and 0.52 for manual and the proposed clipping methods on 28 LGE_Dixon images, and 0.58 and 0.51 for manual and the proposed clipping methods on 7 LGE images, respectively.

**Table 1.** Summary of Dice scores for different segmentation methods (machine learning and traditional methods) and inputs. Italicized values demonstrate the highest performance according to the Dice score.

method and inputs	dataset	clipping method	Dice score of fibrosis segmentation
FK-means; Model M	28 LGE_Dixon	manual	*0.90*
		automatic C1	*0.76*
	7 LGE scans	manual	0.83
		automatic C1	0.72
2D K-means	28 LGE_Dixon	manual	0.54
		automatic C1	0.52
	7 LGE scans	manual	0.58
		automatic C1	0.51
region growing	28 LGE_Dixon	manual	0.66
		automatic C1	0.60
	7 LGE scans	manual	0.66
		automatic C1	0.61

**Figure 5.**
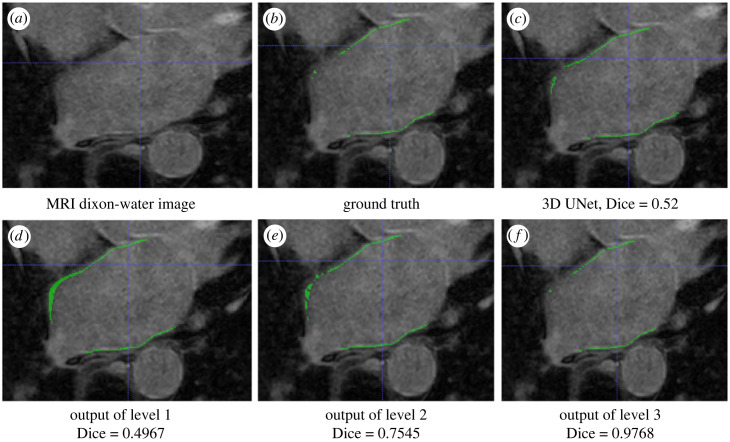
Visualizations of the fibrosis segmentation using models D and M with manual clipping. (*a*) The MRI image. (*b*) Ground truth, with green regions representing fibrosis pixels determined by the valve reference method. (*c*) The results of 3D UNet for fibrosis segmentation. (*d*–*f*) Hierarchical clustering across three levels using K-means clustering notably enhances the Dice score from 0.497 to 0.977.

The visualizations of the fibrosis segmentation are shown in figure 5, which illustrates the differences between the manual segmentation of LA wall and the proposed hierarchical clustering method.

### 4.3. Comparisons with deep leaning-based methods

For the clipping step, a deep learning-based model (3D UNet) was trained for automatic clipping of the MV and PVs using manual segmentation as the training data. The results of clipping method for Clipped_LA, PVs and MV are presented in table 2 to compare the proposed Voronoi-based method and 3D UNet clipping. For the Voronoi-based method, Dice scores of 0.75, 0.80 and 0.73 were obtained for Clipped_LA, PVs and MV, respectively. The implemented 3D UNet achieved a Dice score of 0.74 for Clipped_LA and 0.74 for MV, similar scores to those for the proposed Voronoi-based method. However, the score of 0.63 for PVs suggests relatively lower performance in comparison.

**Table 2.** Dice scores of clipping methods for Clipped_LA, PVs, LAA and MV.

method	Clipped_LA	PVs + LAA	MV
Voronoi-based clipping: model C1	0.75	0.80	0.73
3D UNet clipping: model C2	0.74	0.63	0.74

**Figure 6.**
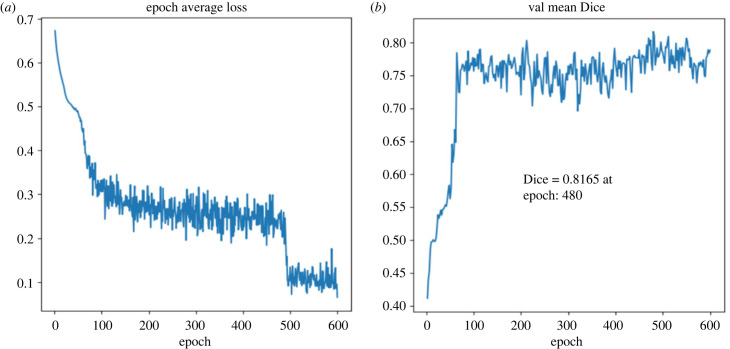
The results of training model. (*a*) The training loss curves over 600 epochs, which shows a consistent decrease in loss over time for training based on manual clipping approach. (*b*) The Dice score over the same epochs. The final Dice score is 0.81.

For fibrosis segmentation, the proposed machine learning-based method was compared with deep learning-based models. Figure 6 provides an overview of the 3D UNet method for fibrosis segmentation, which includes the loss function and Dice score over the training epochs UNet model. The Dice score achieved by the 3D UNet model without clipping on the LA wall was 0.55. However, when incorporating manual clipping, the score improved to 0.80. In comparison, our method, when using manual clipping, achieved a higher Dice score of 0.90. With automatic clipping, the proposed approach still obtained a competitive Dice score of 0.76.

The quantitative results obtained on the test folds of 3D UNet and FK-means are presented in table 3. For the 3D UNet model, threefold cross-validation is performed, and for each fold, the network is trained five times. Table 3 indicates the average scores and standard deviations across these 15 runs. The model with manual clipping achieved high pixel-level scores (precision, recall and Dice index) and low distance-based metrics (ASSD, MSSD, HD, HD95).

**Table 3.** Comparisons of FK-means and the UNet models for fibrosis segmentation based on different clipping models C1, C2 and manual clipping. These are the average scores and standard deviations across 15 runs (threefolds and five runs for each fold).

	precision	recall	average Dice	ASSD	MSSD	HD	HD95
3D UNet models							
without clipping	71.4 ± 0.01	73.6 ± 0.02	55.32 ± 0.01	5.8 ± 1.3	72.4 ± 1.3	43.4 ± 1.5	18.9 ± 1.4
manual clipping	79.1 ± 0.02	84.9 ± 0.04	80.36 ± 0.02	2.6 ± 0.6	55.2 ± 1.0	20.2 ± 1.0	13.4 ± 1.3
Voronoi-based clipping: model C1	73.3 ± 0.03	74.9 ± 0.01	71.00 ± 0.01	5.4 ± 0.5	67.5 ± 5.0	23.5 ± 1.2	14.0 ± 1.5
** **3D UNet clipping: model C2	72.3 ± 0.03	74.2 ± 0.03	69.0 ± 0.03	5.4 ± 1.5	68.1 ± 3.9	30.3 ± 1.3	16.2 ± 1.2
FK-means method							
without clipping	70 ± 0.02	71.34 ± 0.04	0.59 ± 0.02	6.5 ± 0.5	70.5 ± 1.5	38.9 ± 2	15.6 ± 1.3
manual clipping	80.1 ± 0.01	86 ± 0.03	0.90 ± 0.01	1.9 ± 0.6	43.1 ± 1.2	19.2 ± 2	10.0 ± 1.2
Voronoi-based clipping: model C1	79.6 ± 0.03	83.2 ± 0.01	0.76 ± 0.03	5.1 ± 0.2	67.2 ± 2.3	23.7 ± 2.2	14.0 ± 0.5
3D UNet clipping: model C2	64.8 ± 0.02	67.4 ± 0.04	0.64 ± 0.03	6.4 ± 1.5	65.1 ± 3.9	30.4 ± 0.5	14.5 ± 1.3

The Voronoi-based automatic clipping approach (C1) exhibited a slight improvement in the Dice score (71.00 ± 1.8) and demonstrated reduced distance metrics when compared to the deep learning model (C2) for clipping. The results suggest that the performance of deep learning methods and FK-means are rather comparable. However, it appears that the distance metrics and Dice scores for FK-means with manual clipping outperformed all other models.

### 4.4. Repeatability analysis

Table 4 presents the results of the repeatability analysis of segmentations from two different readers (M.H. and I.S.) using the Dice score and ICC on 35 subjects. The analysis was performed on the MV and PVs: the Dice and ICC values indicate that there is moderate to good agreement between the two segmentations for these structures, with slightly higher agreement for MV according to the ICC.

**Table 4.** Repeatability analysis by two readers for the clipping method.

	Dice score	ICC
MV	0.73	0.85
PVs + LAA	0.80	0.83

### 4.5. Parameters analysis

**Figure 7.**
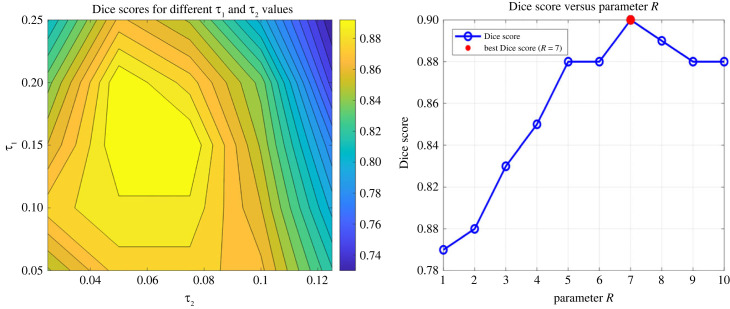
The parameter analysis. Dice score of FK-means for the fibrosis segmentation using parameters *τ*_1_ and *τ*_2_ and *R*. The best Dice score is related to values *τ*_1_ = 0.1 and *τ*_2_ = 0.05 and *R* = 7.

In the method, we employ a combination of adaptive parameter selection and the use of maximum values for certain parameters. The key parameters are adjusted in the K-means clustering, FD and other parameters related to feature extraction. For the FK-means algorithm, three main parameters significantly impact performance, as demonstrated in figure 7.

## 5. Discussion

An automatic FK-means analysis approach for assessment of LA fibrosis from LGE-MRI images was developed and validated. The approach consists of an automatic method for clipping anatomical structures in the LA proximity as well as an automatic fibrosis segmentation method. The proposed automatic clipping method achieved a Dice score of 0.75, similar to the score obtained by a 3D UNet. The proposed automatic fibrosis segmentation method achieved a Dice score of 0.76, slightly higher than the deep learning method (Dice score of 0.69).

Inclusion of MV, LAA and PVs in the LA fibrosis assessment can contribute to reduced accuracy by increased measurement variability and misclassification of non-fibrotic and fibrotic regions. The MV apparatus is intrinsically composed of collagen/fibrosis and inclusion of parts of the MV annulus or leaflet in the segmentation can lead to false positive fibrosis amount [9]. The LAA presents a challenge in segmentation due to its complex geometrical structure with thin walls and trabeculations [8]. Further, the PVs pose difficulties in deciding how much of their structure should be included or excluded in the segmentation process. Excluding the LAA and PVs from the segmentation helps maintain consistency and allows the focus to remain on the LA wall region of interest (ROI) [8].

Accordingly, we propose an automatic clipping method that focuses on accurately segmenting the LA wall edge (Clipped_LA) from LGE-MRI images. Our method incorporates a Voronoi-based method using FD, which enables us to achieve promising results with a Dice score of 0.75 for Clipped_LA segmentation. Furthermore, when combined with manual clipping, our automatic method for fibrosis segmentation yields high performance with a mean Dice score of 0.90. This emphasizes the importance of accurate clipping in achieving precise fibrosis segmentation. The combination of our automatic clipping method with our automatic fibrosis segmentation method achieves a Dice score of 0.76, highlighting the potential value of this combined automatic analysis approach.

In table 5, we compare the performance of our proposed K-means 3D method with the multi-label learning approach by Razeghi *et al*. [9] and the AJSQnet model by Li *et al*. [20]. The multi-label learning approach does not provide Dice scores for fibrosis segmentation, whereas our method achieves a Dice score of 0.76 for this task. Moreover, our method outperforms the multi-label learning approach in PVs segmentation, attaining a Dice score of 0.80 compared to 0.61. Regarding the AJSQnet model, it achieves a Dice score of 0.89 for LA segmentation and 0.58 for fibrosis segmentation, without employing any clipping technique. These results highlight the effectiveness of our proposed method, indicating that the utilization of K-means and Voronoi techniques can provide accurate and reliable segmentation results for anatomical structures and LA fibrosis.

**Table 5.** Comparison of segmentation methods for LA and fibrosis. Italics demonstrate the highest performance according to the Dice score.

method	data	fibrosis segmentation (clustering step)	clipping and truncation (Clipped_LA)	Dice/ICC for fibrosis segmentation
proposed K-means 3D	LGE/LGE-Dixon MRI, 35 manually annotated datasets	hierarchical K-means method	Voronoi-based method	*Dice = 0.76*
multi-label learning [9]	registration CE-MRA and LGE-MRI, 207 manually annotated datasets	threshold-based method	CNN network	ICC = 0.99
AJSQnet model [20]	MICCAI2018 and ISBI2012, LGE-MRI	deep learning method	—	Dice = 0.58
current implementation of 3D UNet	LGE_Dixon MRI, 28 manually annotated datasets	deep learning method	3D UNet	Dice = 0.69

While deep learning methods show promise for fibrosis segmentation, they have limitations such as the requirement for large amounts of training data and computational resources. Additionally, the lack of interpretability and transparency in deep learning models can make it difficult to understand how the model arrived at its decisions, which can have significant implications in medical imaging. By contrast, traditional machine learning and classification-based methods may provide more transparent and robust results, particularly in situations with limited data.

The proposed method introduces hierarchical clustering, which sets it apart from K-means and 3D K-means++. This hierarchical approach allows for clustering at different levels, accommodating varying numbers of clusters and enabling detailed analysis and classification of fibrosis pixels based on texture characteristics. Furthermore, the method incorporates FD as a feature in the distance metric, while K-means and 3D K-means++ rely solely on the Euclidean distance. Additional enhancements include penalty terms, automatic parameter tuning, and stopping metrics, which improve clustering performance and efficiency. The results demonstrate that the fractal-based K-means++ algorithm achieved a Dice coefficient of 0.76, outperforming the K-means++ algorithm with a Dice coefficient of 0.65. This indicates that the inclusion of FD calculation as a penalty term improved the accuracy of identifying and delineating fibrotic regions.

In this approach, FD is considered as pixel-level texture features, prioritizing texture characteristics over the global complexity within the ROI. A larger value for the crucial parameter in FD estimation captures global structural complexity, while values *R* < 7, with a scaling factor of 2 < *r* < *R*, emphasize local fibrosis-related details. FK-means demonstrates robustness, consistently delivering effective results across 4769 images from two different image modalities, with the aim of reducing under and over FD estimation. Tackling FD estimation challenges involves a twofold strategy: (i) incorporating adaptive scaling via Gaussian mixture models for precise global and local feature extraction and (ii) integrating CNNs to simplify parameters and enhance feature extraction for accurate fibrosis segmentation.

The primary challenges in machine learning and deep learning often revolve around the problem of overfitting. In the context of FK-means, overfitting is largely associated with the selection of the optimal number of clusters. However, the adoption of a hierarchical K-means approach within FK-means alleviates the necessity for manual cluster selection. This innovative approach not only enhances the precision of fibrosis segmentation but also effectively addresses concerns related to overfitting. Leveraging a substantial dataset comprising 4769 images, this methodology proves to be highly effective in mitigating overfitting challenges. Additionally, for deep learning techniques, we have implemented various data augmentation strategies to further combat the issue of overfitting.

Nonetheless, it is essential to recognize that data limitations and the need for manual segmentation remain noteworthy challenges in this project to mitigate overfitting and FD estimation issues and improve the training models. However, the advantage of the proposed machine learning-based K-means model is its ability to perform well with a relatively small dataset. To improve segmentation accuracy, future work could explore larger datasets and investigate 3D UNet models in this current study, which have shown promising results in various medical imaging tasks. Additionally, our proposed method assumes clear geometric characteristics of the LA, PVs and MV, which may not be accurate in all cases. It also relies on accurate initial LA wall segmentation, which can be challenging in cases with thin or irregular borders. Future research could focus on addressing these limitations by developing robust algorithms that can handle variations in anatomical structures and adapt to different imaging conditions.

In conclusion, the current findings suggest that FK-means enables reliable LA fibrosis segmentation. This performance in combination with the transparency and interpretability of the presented method makes it a promising tool for the assessment of atrial fibrosis. The data also suggest that clipping of anatomical structures in the atrial proximity can add to the segmentation of LA fibrosis.

## Data Availability

The code and data supporting this study are available from LiU Secure Repository at: https://doi.org/10.48360/m803-yp37 [24].
